# A systematic review and meta-analysis of the relationship between magnesium levels and malaria severity

**DOI:** 10.1038/s41598-024-51718-z

**Published:** 2024-01-16

**Authors:** Kwuntida Uthaisar Kotepui, Aongart Mahittikorn, Polrat Wilairatana, Frederick Ramirez Masangkay, Manas Kotepui

**Affiliations:** 1 Medical Technology, School of Allied Health Sciences, Walailak University, Thasala, Nakhon Si Thammarat, 80160 Thailand; 2 Department of Protozoology, Faculty of Tropical Medicine, Mahidol University, Bangkok, 10400 Thailand; 3 Department of Clinical Tropical Medicine, Faculty of Tropical Medicine, Mahidol University, Bangkok, 10400 Thailand; 4 Department of Medical Technology, Faculty of Pharmacy, University of Santo Tomas, 1008 Manila, Philippines

**Keywords:** Parasitology, Malaria

## Abstract

Magnesium is associated with *Plasmodium* infections and malaria severity. This systematic review and meta-analysis was conducted to synthesize the link between *Plasmodium* infections and magnesium levels for improved clinical guidance and therapeutic interventions in malaria-affected regions. A systematic literature search was conducted across multiple databases, including ProQuest, Scopus, Embase, Ovid, MEDLINE, PubMed, and Google Scholar. The risk of bias in the selected studies was assessed using the Joanna Briggs Institute critical appraisal tools. A thematic synthesis was employed to demonstrate the magnesium levels across selected studies, for analyzing and grouping based on geographic regions, age demographics, and clinical manifestations of malaria. Meta-analyses determined differences in magnesium levels between individuals with malaria, uninfected controls, and patients with different clinical severities of malaria. The effect sizes from individual studies were pooled using the random-effects model. Out of 2533 records identified, 13 studies were included in the review. The thematic synthesis revealed complex and varied results, with studies showing different magnesium levels in malaria patients across different geographies, age groups, and clinical presentations. The meta-analysis indicated elevated magnesium levels in malaria patients compared with uninfected controls (*P* < 0.01, Hedges’ g: 1.94, 95% CI 0.86–3.03, *I*^2^: 98.38%, 9 studies). No statistically significant difference was observed in magnesium levels between patients with severe and nonsevere malaria (*P*: 0.34, Hedges’ g: 0.62, 95% CI − 0.64–1.88, *I*^2^: 91.46%, 2 studies). A significant increase in magnesium levels was seen in patients with malaria who died compared with those who survived (*P* < 0.01, Hedges’ g: 0.39, 95% CI 0.13–0.64, *I*^2^: 3.39%, 3 studies). This systematic review and meta-analysis presented relationship between magnesium levels and malaria. While the meta-analysis indicated a general trend of increased magnesium levels in patients with malaria, the substantial heterogeneity and instability of the results hint toward a rich yet uncharted territory requiring more research depth. The intricate interplay between magnesium levels and malaria beckons a multidimensional approach in future studies.

## Introduction

Malaria, a mosquito-borne infectious disease caused by *Plasmodium* parasites, poses a significant global health challenge^1^. This disease is most prevalent in tropical and subtropical regions, including parts of Sub-Saharan Africa, Asia, and the Americas and affects millions of people each year^2^. The life cycle of the *Plasmodium* parasite, involving the *Anopheles* mosquito and humans, leads to the characteristic symptoms of malaria, including fever, headache, and other flu-like symptoms^3^. If untreated promptly, the disease can progress to severe forms, leading to complications and even death^4,5^.

Micronutrients, vitamins, and minerals required in small quantities are pivotal in maintaining optimal health and metabolic integrity^6^. They assist in various physiological functions, from immune defense and DNA repair to energy production and neural development^6^. Micronutrients deficiency can have harmful effects, leading to various health disorders and diseases^7^. The relationship between micronutrient status and infectious diseases is intricate, as infections can influence micronutrient absorption and metabolism. In contrast, the presence or absence of certain micronutrients can impact an individual’s susceptibility to infections^8–11^.

Magnesium, a vital mineral and one of the essential micronutrients is crucial for numerous biological processes within the human body, such as proper muscle, nerve, and enzyme function^12^. Magnesium acts as cofactor for over 300 enzymatic reactions and plays crucial role in energy production, DNA replication, protein synthesis, and neuromuscular transmission^12,13^. Despite its significance, magnesium deficiencies are common, especially in populations with limited dietary diversity. Such deficiencies can manifest as various symptoms, including muscle cramps, skeletal deformities, cardiovascular diseases, and metabolic syndromes^12,14,15^. Recent studies have indicated a possible connection between malaria and magnesium levels, observed in in vitro and animal studies^16–19^. A study reported that adding magnesium chloride to the culture medium of *P. vivax* enhanced the differentiation of schizonts into merozoites^16^. In mouse models infected with *P. chabaudi*, magnesium deficiency correlated with lower erythrocyte magnesium levels and reduced parasitemia^19^.

Additionally, a separate study found that mice infected with *P. berghei* with high plasma magnesium levels had significantly longer survival time^18^. Furthermore, human studies have observed altered magnesium levels in individuals with malaria, indicating a possible interplay between the parasite’s life cycle and magnesium metabolism^20–22^. This association is interesting as it offers insights into the pathophysiological changes induced by malaria and could have implications for patient care and treatment strategies.

Understanding the relationship between malaria and magnesium levels is essential, for potential implications for patient prognosis and treatment. Although several studies have explored this association, the results have remained inconsistent, often due to varying methodologies, populations, or geographical factors^20–23^. A systematic review and meta-analysis would provide a consolidated perspective by synthesizing the existing evidence, increasing the overall statistical power, and addressing interstudy variability. Such an analysis would offer clearer insights into the association between malaria severity and magnesium levels, help identify gaps in current knowledge, and set the stage for standardized and methodologically sound future studies. This endeavor is promising for improved clinical guidance and potentially transformative therapeutic interventions in malaria-affected regions.

## Method

### Protocol and registration

This systematic review was registered with PROSPERO (CRD42023464691). Comprehensive and transparent reporting was ensured by adhering to the Preferred Reporting Items for Systematic Reviews and Meta-Analyses (PRISMA) guidelines^24^.

### A systematic review of questions

A systematic review question was developed using the PECO framework: P is Population, E is Exposure, C is Comparator, and O is outcome^25^. A systematic review question was framed as “In children and adults residing in malaria-endemic areas (P), how does *Plasmodium* infection (E) compare to uninfected controls, less severe malaria cases, or survivor cases (C) in terms of its impact on magnesium levels (O)?”.

### Outcomes of the systematic review and meta-analysis

There were three outcomes in this study: (i) the difference in magnesium levels between individuals with and without malaria (uninfected controls); (ii) the difference in magnesium levels between severe and nonsevere malaria cases; and (iii) the difference in magnesium levels between patients who died from malaria and who survived.

### Search strategy and selection criteria

A systematic literature search was conducted across multiple databases, including ProQuest, Scopus, Embase, Ovid, MEDLINE, and PubMed (Table S1) with an objective to identify studies evaluating magnesium levels in relation to malaria. Studies were excluded if they were unrelated to the targeted participants, not pertinent to the desired outcome, conference abstracts, in vitro studies, reviews, animal studies, case reports, or case series. Additionally, studies that reported magnesium levels exclusively in malaria cases without uninfected controls or only in one specific group of malaria cases without comparing disease severity were excluded. In addition, complementary searches on Google Scholar and reference list checks of relevant articles were conducted to ensure the comprehensiveness of the searches.

For the study selection process, duplicates were removed, and the remaining studies underwent a two-stage process: initial screening based on titles and abstracts, followed by full-text assessment for eligibility against the inclusion criteria. If the full text was unavailable, the authors were contacted via ResearchGate (https://www.researchgate.net/) for full-text copies request. Two independent authors conducted the study selection process. Discrepancies or disagreements that arose regarding the inclusion or exclusion of particular studies were cleared through a thorough discussion to reach a consensus. A third reviewer was consulted to make the final decision if a consensus could not be reached through discussion.

### Data extraction and quality assessment

The following characteristics of the included studies were extracted in an Excel spreadsheet (Microsoft Corporation, Redmond, WA): publication year, study design, study location, specific focus on *Plasmodium* species, participant demographics, methods of *Plasmodium* species detection, and methods and blood samples for magnesium determination. The risk of bias in the selected studies was assessed using the Joanna Briggs Institute critical appraisal tools^26^. The tools evaluate aspects such as inclusion criteria and statistical analysis for cross-sectional studies. Cohort studies are appraised based on group similarities, exposure measurements, and handling of confounding factors. Case–control studies are assessed based on case/control appropriateness, exposure measurement, and management of confounding factors. The criteria for randomized controlled trials focus on randomization processes, group similarities, blinding, and follow-up strategies. Each criterion is posed as a question, with potential answers like “Yes,” “No,” or “Unclear,” providing insight into the study’s overall quality. Two independent authors performed the data extraction and quality assessment processes. When there were discrepancies or disagreements, a third reviewer was consulted to make the final decision.

### Data synthesis and analysis

For the thematic synthesis^27^, the magnesium levels across selected studies were analyzed and grouped based on geographic regions, age demographics, and clinical manifestations of malaria. Meta-analyses were performed to assess the differences in magnesium levels between individuals with malaria and uninfected controls and among patients with different clinical severities of malaria. Hedges’ g was used as the effect size measure, and the effect sizes from individual studies were pooled using the random-effects model based on the DerSimonian and Laird methods^28^. Heterogeneity was evaluated using the *I*^2^ statistic. An *I*^2^ value of 0% suggests no observed heterogeneity; 25% is seen as low, 50% as moderate, and 75% as high^29^. Metaregression and subgroup analyses were performed to explore potential sources of heterogeneity in the data. The metaregression analysis was undertaken only when a minimum of 6–10 studies were included in the meta-analysis^30^. Factors considered in the metaregression and subgroup analyses included publication year, age group, *Plasmodium* species, diagnostic method for malaria, and blood samples for measuring magnesium. The results of the meta-analysis were visualized through the forest plot. A publication bias was detected using a funnel plot if the number of studies included in the meta-analysis was at least 10^31^. A leave-one-out sensitivity analysis was conducted to test the robustness of the meta-analysis results by recalculating the overall effect estimate while excluding one study at a time^32^. All statistical evaluations were performed using Stata 18.0 software (StataCorp LLC, College Station, TX).

## Results

### Search results

**Figure 1:**
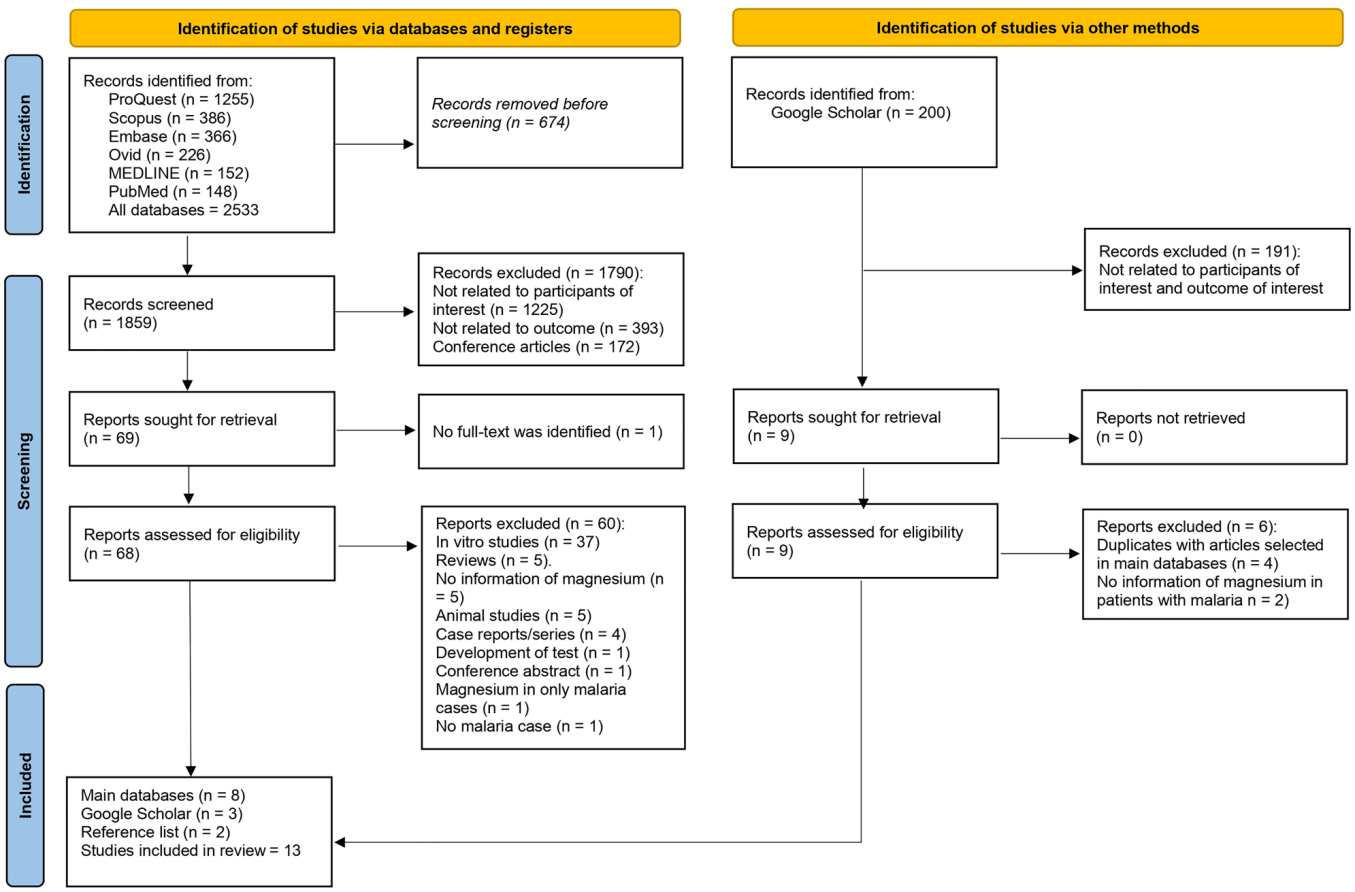
The study flow diagram demonstrated the selection of studies.

Overall, 2533 records were identified from several databases, including ProQuest (n = 1255), Scopus (n = 386), Embase (n = 366), Ovid (n = 226), MEDLINE (n = 152), and PubMed (n = 148). Before the screening phase, 674 duplicate records were removed, resulting in 1859 records for screening. During the screening process, 1790 records were excluded for the following reasons: not related to the participants of interest (1225 articles), not pertinent to the outcome of interest (393 articles), and being conference articles (172 articles). In total, 69 reports were selected for further assessment. However, one study could not be retrieved because the full text was unavailable, leading to 68 reports being assessed for eligibility. In the assessment phase for eligibility, out of the 68 studies scrutinized, 60 were subsequently excluded for several reasons, mainly in vitro studies. Eight studies from the main databases were considered eligible^20–23,33–36^. Additional resources were utilized, including Google Scholar and a review of reference lists from studies in the main databases. Screening of 200 Google Scholar records led to excluding 191 articles unrelated to the participants or outcomes of interest. Further full-text review resulted in the exclusion of six articles, while three articles met the inclusion criteria^37–39^. Additionally, reviewing the reference lists from the main databases yielded two more studies that fit the inclusion criteria^40,41^. In total, 13 studies were deemed suitable for inclusion in the review^20–23,33–41^ (Fig. 1).

### Characteristics of the included studies

The 13 studies reviewed were primarily published from 2010 to 2019 (53.85%) and employed cross-sectional (53.85%). Most were conducted in Africa (61.54%), notably Nigeria (30.77%), and Asia (38.46%), predominantly Pakistan (15.38%). The primary focus was on *P. falciparum* species (76.92%) and adult participants (53.85%). While the majority addressed symptomatic malaria (53.85%), there was a varied representation of malaria severity, with many not specifying disease severities (46.15%). *Plasmodium* species detection methods were diverse, including microscopic methods (46.15%) and a combination of microscopic and rapid diagnostic tests (RDTs) (23.08%). In contrast, magnesium determination was primarily performed through a variety of methods (61.54%) using both serum (53.85%) and plasma (46.15%) samples (Table 1, Table S2).

**Table 1:** Summary features of the studies included in the review.

Characteristics	n. (13 studies)	%
**Publication years**		
Before 2000	1	7.69
2000–2009	4	30.77
2010–2019	7	53.85
2020–2023	1	7.69
**Study designs**		
Cross-sectional study	7	53.85
Case–control study	4	30.77
Cohort study	1	7.69
Clinical trials	1	7.69
**Study areas**		
Asia	**5**	38.46
Pakistan	2	15.38
India	1	7.69
Thailand	1	7.69
Vietnam	1	7.69
Africa	**8**	61.54
Nigeria	4	30.77
Sudan	1	7.69
Cameroon	1	7.69
Kenya	1	7.69
Tanzania	1	7.69
***Plasmodium*** **species**		
*P. falciparum*	10	76.92
*P. vivax*	1	7.69
Not specified	2	15.38
**Participants**		
Children	4	30.77
Adults	7	53.85
Children and adults	1	7.69
Not specified	1	7.69
**Symptoms**		
Symptomatic	7	53.85
Asymptomatic	1	7.69
Not specified	5	38.46
**Severity status**		
Severe malaria	2	15.38
Severe and nonsevere malaria	2	15.38
Nonsevere malaria (uncomplicated, mild, or asymptomatic malaria)	2	15.38
Asymptomatic	1	7.69
Not specified	6	46.15
**Methods for malaria detection**		
Microscopic method	6	46.15
Microscopic method, RDT	3	23.08
Not specified	4	30.77
**Methods for magnesium determination**		
Atomic absorption spectrometry	5	38.46
Other methods	8	61.54
**Blood sample for magnesium measurement**		
Serum	7	53.85
Plasma	6	46.15

RDT, rapid diagnostic test.

### Risk of bias of the included studies

For cross-sectional studies, one study was unclear, and no strategies for handling confounding factors were outlined to address them^22^. Similarly, three studies^20,21,35^ did not identify or state strategies for confounding factors but satisfied all other checkpoints. In contrast, three studies^23,34,39^ comprehensively met all the criteria, including identifying and formulating strategies to handle confounding factors. For case–control studies, four studies^33,37,38,41^ shared common shortcomings. Besides the presence or absence of disease, the comparability of groups remained unclear in all studies, and confounding factors were neither identified nor strategies devised to address them. The appropriateness of case and control matching was unclear in the two studies^37,41^, whereas two studies^33,38^ successfully matched the cases and controls appropriately, enhancing the robustness of their studies. The cohort study did not clearly indicate whether the follow-up time was sufficiently long for the outcomes to occur. Similarly, the completeness of the follow-up and the strategies employed to address any incomplete follow-ups were not explicitly stated^40^. The clinical trial study demonstrated areas of uncertainty, as several criteria were marked “unclear,” raising questions about potential biases related to concealment of treatment allocation, blinding of those delivering treatment, and completeness of participant follow-up, among others. Notably, participants were not blinded to the treatment assignments^36^ (Table S3).

### Thematic synthesis of magnesium levels in malaria

Several studies, including those by Baloch et al.^38^, Garba et al.^35^, Oluboyo et al.^41^, and Okon et al.^20^, reported increased magnesium levels, while, studies by Abdelsalam et al.^22^ and Baloch et al.^33^ noted decreased adenosine aminotransferase levels. Meanwhile, Asaolu et al.^37^ and Mbugi et al.^23^ found no substantial changes in magnesium levels. The studies analyzed offer a detailed view of the relationship between magnesium levels and malaria, unearthing diverse outcomes across different geographic regions and age groups. Studies based on African countries, such as those by Abdelsalam et al.^22^, observed a decrease, whereas Asaolu et al.^37^ found no significant change. In contrast, the Asian-centric studies by Baloch et al.^38^ and Dondorp et al.^40^ reported an increase in magnesium levels, showcasing a varied landscape in the understanding of this relationship globally.

Delving into the age demographics highlighted in studies accentuates the intricate interplay between magnesium levels and malaria. The adult population has been at the center of conflicting results, with a discrepancy between increased and decreased magnesium levels being reported in several studies. Meanwhile, studies focused on children have been considerable, with studies such as those by Maitland et al.^36^ and Mbugi et al.^23^ focusing on the distinct magnesium level trends in younger individuals, ranging from no noticeable difference to significant alterations during malaria infections. Different trends based on the severity and symptomatology of malaria were observed while analyzing the clinical manifestations of malaria. Davis et al.^34^ and Mfonke et al.^39^ highlighted these magnesium level fluctuations, revealing differences between severe and nonsevere malaria cases. In addition, the study by Mbugi et al.^23^ enrolled both participants with symptomatic and asymptomatic malaria, emphasizing that the trends in magnesium levels have substantial fluctuations, mainly in symptomatic cases where the levels can increase or decrease markedly. Davis et al.^34^ and Oluboyo et al.^41^ revealed a focused view on fatal cases, which provided evidence that magnesium levels can either significantly increase or remain unchanged in fatal malaria cases (Table 2).

**Table 2:** Comparison of magnesium levels in patients with malaria.

No.	Authors	Study location	*Plasmodium* spp.	Age range (years)	Clinical malaria (severe, uncomplicated, mild)	Clinical malaria (symptomatic or asymptomatic	Magnesium levels in patients with malaria
1	Abdelsalam et al.^22^	Sudan	*P. falciparum*	Adults (≥ 18 years)	Not specified	Symptomatic malaria	1. Significantly decreased levels of magnesium were observed in patients with malaria when compared to controls. 2. Magnesium levels were decreased with increasing parasitaemia
2	Asaolu et al.^37^	Nigeria	*P. falciparum*	20–43 years	Not specified	Not specified	No difference in levels of magnesium was observed in patients with malaria when compared to controls
3	Baloch et al.^38^	Pakistan	*P. falciparum*	Not specified	Not specified	Not specified	Significantly increased levels of magnesium were observed in patients with malaria when compared to controls
4	Baloch et al.^33^	Pakistan	*P. vivax*	Not specified	Not specified	Not specified	Significantly decreased levels of magnesium was observed in patients with malaria when compared to controls
5	Davis et al.^34^	Thailand	*P. falciparum*	14–72 years	Severe and non-severe malaria	Symptomatic malaria	1. Significantly increased levels of magnesium were observed in patients who died when compared to survivors. 2. No difference in levels of magnesium was observed in patients with severe malaria when compared to non-severe malaria
6	Dondorp et al.^40^	Vietnam	*P. falciparum*	Fatal cases (46): 15–74, survivors (222): 15–79	Severe malaria	Symptomatic malaria	Significantly increased levels of magnesium were observed in fatal cases when compared to survivors
7	Garba et al.^35^	Nigeria	*P. falciparum*	18–40 years	Non-severe malaria	Symptomatic malaria	Significantly increased levels of magnesium were observed in patients with malaria when compared to controls
8	Maitland et al.^36^	Kenya	*P. falciparum*	> 2 months	Severe malaria	Symptomatic malaria	No difference in magnesium levels was observed between fatal cases and survivors
9	Mbugi et al.^23^	Tanzania	*P. falciparum*	6–72 months	Asymptomatic malaria	Asymptomatic malaria	No difference in magnesium deficiency was observed in patients with malaria when compared to controls
10	Mfonke et al.^39^	Cameroon	*P. falciparum*	Not specified	Severe and non-severe malaria	Symptomatic malaria	Significantly decreased levels of magnesium were observed in patients with malaria (uncomplicated, malaria anemia, cerebral malaria) when compared to controls
11	Okon et al.^20^	Nigeria	*P. falciparum*	Not specified	Not specified	Not specified	1. Significantly increased levels of magnesium were observed in patients with malaria when compared to controls. 2. Significantly increased levels of magnesium were observed in patients with severe malaria when compared to mild malaria. 3. Significantly increased levels of magnesium were observed in patients with moderate malaria when compared to mild malaria. 4. No difference in magnesium was observed between moderate and severe malaria
12	Oluboyo et al.^41^	Nigeria	Not specified	18–22 years	Not specified	Not specified	Significantly increased levels of magnesium were observed in patients with malaria when compared to controls
13	Tyagi et al.^21^	India	Not specified	13–82 years	Non-severe malaria	Symptomatic malaria	Significantly decreased levels of magnesium were observed in patients with malaria when compared to controls

### Meta-analysis of differences in magnesium levels between malaria- and uninfected individuals

**Figure 2:**
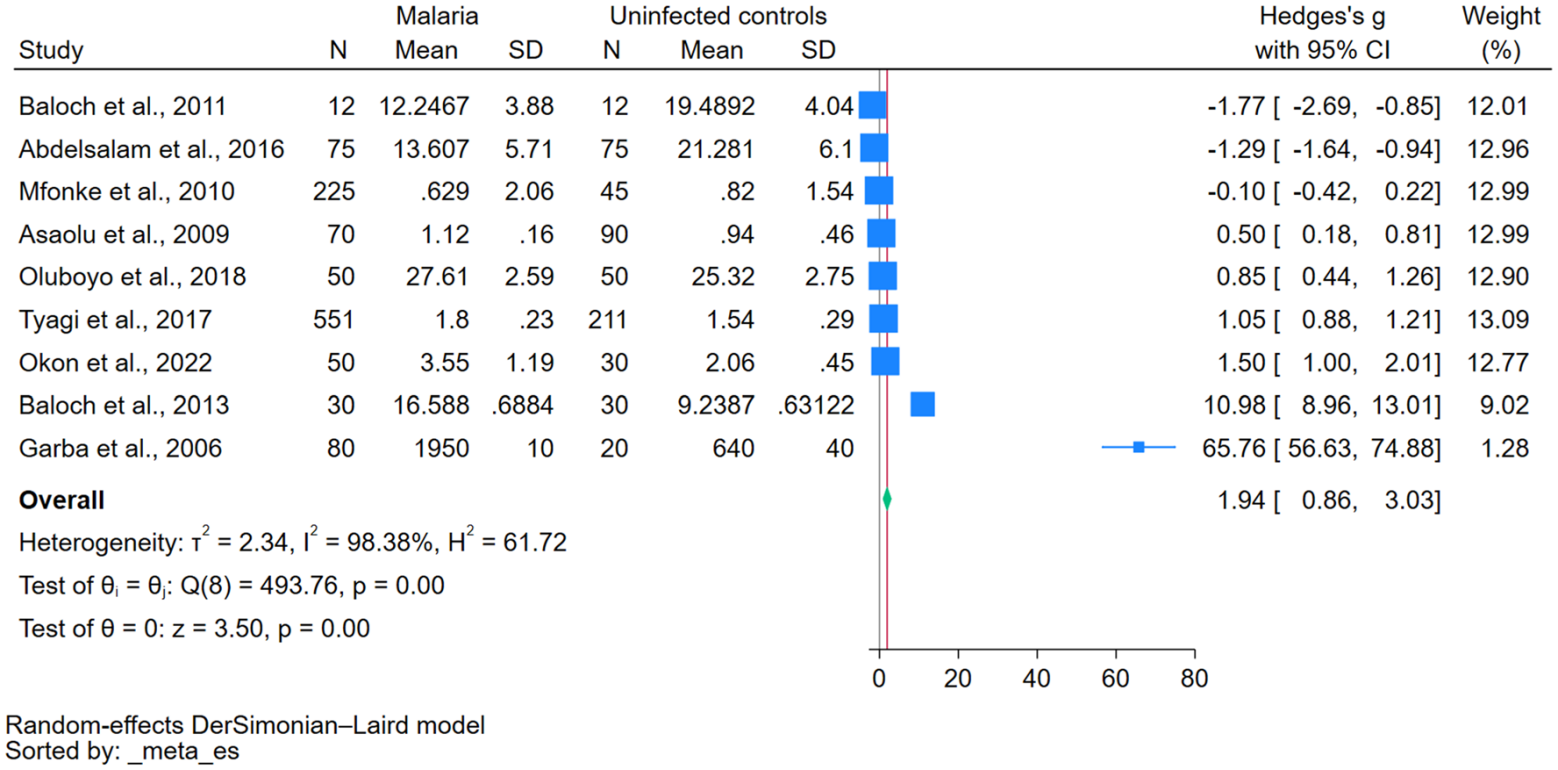
The forest plot demonstrated a significant elevation of magnesium levels in patients with malaria compared with uninfected controls. Blue square, effect estimates of individual studies; green diamond, pooled effect estimate; red horizontal line, pooled effect estimate; gray horizontal line, no effect line; N, number of populations in each group; CI, confidence interval; SD, standard deviation.

The differences in magnesium levels between individuals with malaria and uninfected individuals were analyzed using data from nine studies^20–22,33,35,37–39,41^. The results indicated elevated magnesium levels in patients with malaria compared with uninfected controls (*P* < 0.01, Hedges’ g: 1.94, 95% confidence interval (CI) 0.86–3.03, *I*^2^: 98.38%, 9 studies, Fig. 2). Given the substantial heterogeneity in the meta-analysis results (*I*^2^: 98.38%), metaregression and subgroup analyses were performed to identify the potential sources of this heterogeneity. The metaregression analysis revealed that factors such as publication year, age group, *Plasmodium* species, the diagnostic method for malaria, and the method for measuring magnesium significantly affected the pooled estimate (*P* < 0.05, see Table S4).

The subgroup meta-analysis in Table 3 revealed mixed results regarding the differences in magnesium levels across various criteria. Although no consistent trends were observed during different publication periods and among different study designs, geographic discrepancies came to the fore; significant differences were evident in African studies (*P* = 0.02) in contrast to those conducted in Asia (*P* = 0.11). Parsing the data by age groups highlighted notable discrepancies in magnesium levels among adults (*P* = 0.03) compared to those among children (*P* = 0.39). A breakdown based on the identified *Plasmodium* species revealed significant differences, especially in studies involving *P. falciparum* (*P* < 0.01). Moreover, the methodology employed for *Plasmodium* detection and magnesium determination had substantial influence; studies using the microscopic method for *Plasmodium* detection (*P* < 0.01) and the atomic absorption spectrometry for magnesium determination (*P* < 0.01) displayed significant differences in magnesium levels. Finally, a comparative analysis of blood sample types underscored marked differences in magnesium levels, particularly in serum samples (*P* < 0.01).

**Table 3:** Subgroup analyses of differences in magnesium levels between patients with malaria and uninfected controls.

Subgroup analyses	*P* value	Hedges’ g (95% CI)	*I*^2^ (%)	Number of studies
**Publication years**				
2020–2023	N/A	1.50 (0.03–3.02)	N/A	1
2010–2019	0.07	1.10 (− 0.07–2.27)	98.24	6
2000–2009	0.31	32.96 (− 30.99–96.91)	99.49	2
**Study designs**				
Cross-sectional study	0.01	2.19 (0.53–3.84)	98.91	5
Case–control study	0.02	2.18 (0.30–4.06)	97.66	4
**Continents**				
Africa	0.02	1.71 (0.29–3.14)	98.38	6
Asia	0.11	3.24 (− 0.73–7.21)	98.44	3
**Age groups**				
Children	0.39	0.69 (− 0.88–2.26)	96.36	2
Adults	0.03	2.27 (0.26–4.28)	98.63	5
Children and adults	N/A	1.05 (0.88–1.21)	N/A	1
Not specified	N/A	10.98 (8.96–13.01)	N/A	1
***Plasmodium*** **species**				
*P. falciparum*	< 0.01	4.18 (2.32–6.04)	98.77	6
*P. vivax*	N/A	− 1.77 (− 2.69–(− 0.85))	N/A	1
Not specified	< 0.01	1.02 (0.86–1.17)	0	2
**Symptoms**				
Symptomatic	0.01	2.73 (0.70–14.77)	99.15	4
Not specified	0.01	1.93 (0.50–3.37)	97.10	5
**Methods for malaria detection**				
Microscopic method	< 0.01	2.19 (0.89–3.49)	98.07	5
Microscopic method, RDT	0.25	− 0.69 (1.86–0.48)	95.91	2
Not specified	0.47	4.58 (− 7.92–17.07)	99.21	2
**Methods for magnesium determination**				
Atomic absorption spectrometry	< 0.01	6.80 (3.96–9.63)	98.76	5
Other methods	0.63	0.29 (− 0.86–1.43)	98.23	4
**Types of blood samples**				
Serum	< 0.01	3.58 (1.78–5.38)	98.61	7
Plasma	0.40	0.48 (− 0.64–1.60)	97.41	2

CI, confidence interval; N/A, not assessed; RDT, rapid diagnostic test.

### Meta-analysis of differences in magnesium levels in relation to clinical severity

**Figure 3:**
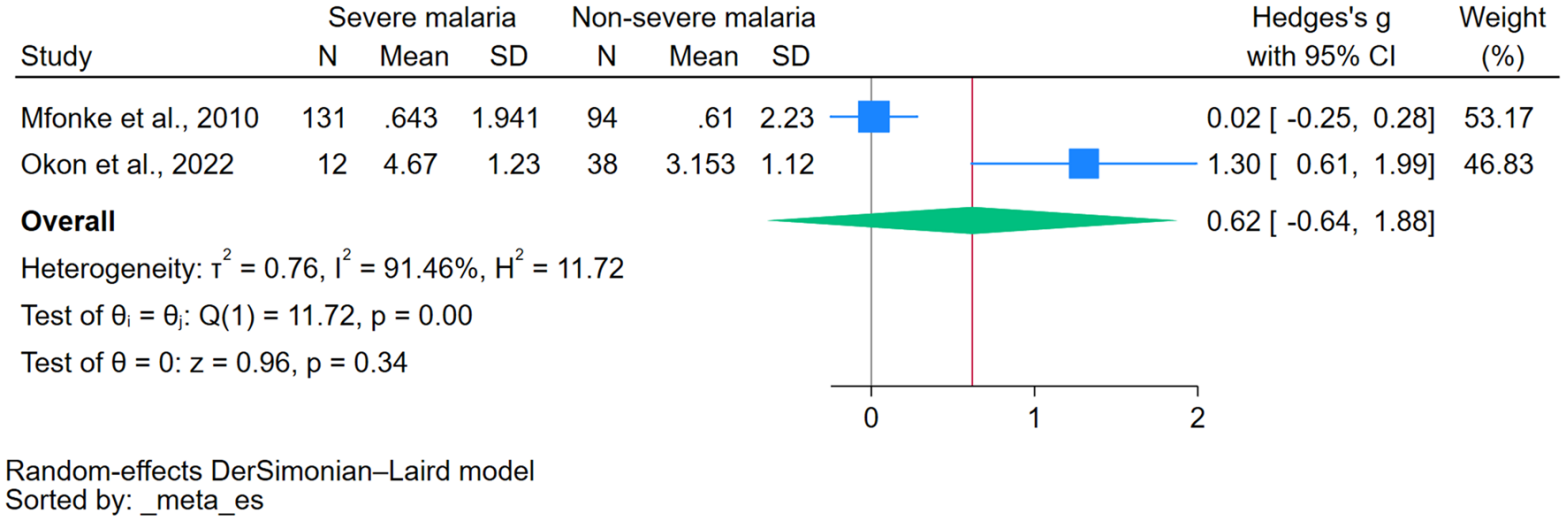
The forest plot demonstrated a significant elevation of magnesium levels in patients with severe malaria compared to those with nonsevere malaria. Blue square, effect estimates of individual studies; green diamond, pooled effect estimate; red horizontal line, pooled effect estimate; gray horizontal line, no effect line; N, number of populations in each group; CI, confidence interval; SD, standard deviation.

**Figure 4:**
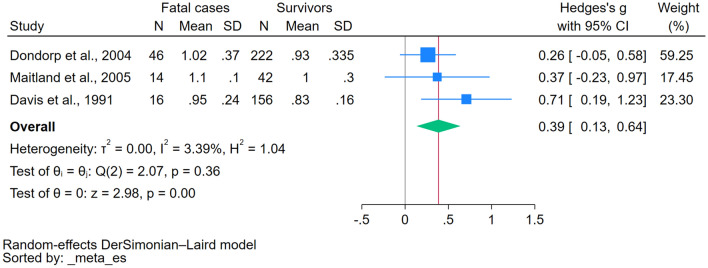
The forest plot demonstrated a significant elevation of magnesium levels in patients with fatal malaria compared with survivors. Blue square, effect estimates of individual studies; green diamond, pooled effect estimate; red horizontal line, pooled effect estimate; gray horizontal line, no effect line; N, number of populations in each group; CI, confidence interval; SD, standard deviation.

The difference in magnesium levels between patients with severe and nonsevere malaria was estimated using the data from two studies^20,39^. The analysis revealed no statistically significant disparity in magnesium levels between the two groups (*P*: 0.34, Hedges’ g: 0.62, 95% CI − 0.64–1.88, *I*^2^: 91.46%, 2 studies, Fig. 3). The difference in magnesium levels between patients with malaria who died and those who survived was estimated using data from three studies ^34,36,40^. There was a significant increase in magnesium levels in patients with malaria who died than those who survived (*P* < 0.01, Hedges’ g: 0.39, 95% CI 0.13–0.64, *I*^2^: 3.39%, 3 studies, Fig. 4).

### Publication bias and sensitivity analysis

**Figure 5:**
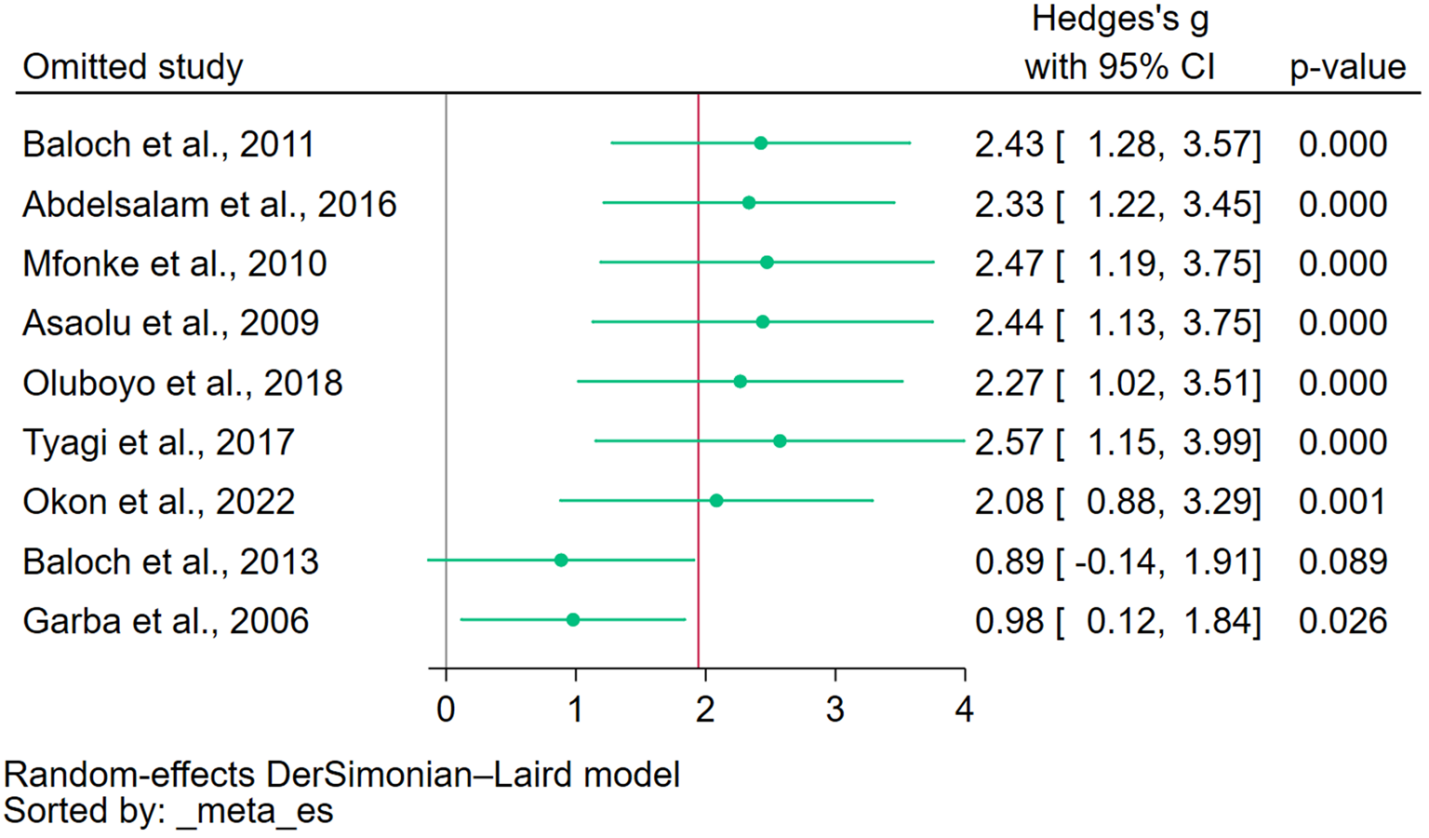
The leave-one-out meta-analysis demonstrated how stable the analysis was when each study was excluded from the meta-analysis and the analysis was rerun. Green dots, pooled effect estimate; red vertical line, overall effect estimate; gray vertical line, no effect line; green vertical line, confidence interval; CI, confidence interval.

An assessment of publication bias was not conducted because the number of studies included in the meta-analysis was less than 10, a threshold commonly set to maintain the statistical power and reliability of the results. However, a leave-one-out meta-analysis of the difference in magnesium levels between patients with malaria and uninfected controls was undertaken to scrutinize the robustness of the meta-analysis findings. This approach revealed instability in the meta-analysis outcomes, where the exclusion and subsequent reanalysis with each study individually omitted led to fluctuating results, demonstrating a lack of consistent patterns across the studies (Fig. 5). This suggests that the conclusions drawn from this meta-analysis should be interpreted cautiously, considering the potential for substantial alterations in the outcomes with slight modifications in the dataset.

## Discussion

This systematic review investigated the relationship between *Plasmodium* infections and magnesium levels, explicitly focusing on alterations in magnesium metabolism. The studies in the systematic review presented diverse outcomes regarding magnesium levels in patients with malaria. While several studies reported elevated magnesium levels in patients with malaria, indicating a potential disruption in magnesium homeostasis linked to the disease’s progression or severity^20,35,38,41^, others found no significant difference in magnesium levels compared with noninfected individuals^23,37^. Additionally, some studies observed decreased magnesium levels^22,33^, suggesting a possible depletion or altered use of magnesium in response to the infection. The geographical distinctions are compelling, with studies from Africa presenting a discrepancy in magnesium levels, ranging from decreased to no significant change. In contrast, Asian studies consistently reported increased magnesium levels in patients with malaria. These variations indicate a potential interaction between regional factors, such as nutritional or genetic factors^42–45^. A clear distinction in age demographics emerges between adults and children regarding magnesium levels during malaria infections. Adults showed a significant increase in magnesium levels, whereas children exhibited no discernible change. This disparity might point to a developmental factor that affects the body’s metabolic response to malaria, highlighting the need for age-specific studies to delve deeper into this phenomenon. It is worth noting that magnesium levels in the intracellular compartment tend to decrease with age^46^. Furthermore, serum magnesium levels are lower in the elderly due to inadequate intake, impaired intestinal absorption, or renal wasting^47^.

Subgroup analysis based on *Plasmodium* species revealed elevated magnesium levels in patients with *P. falciparum* malaria compared with uninfected controls. However, because only one study considered patients with *P. vivax* malaria, the impact of different *Plasmodium* species on magnesium levels in malaria patients remains to be determined. Studies have delved deep into the clinical manifestations, unearthing trends in magnesium levels contingent on the severity and symptomatology of malaria. Subgroup analysis revealed no significant increase in magnesium levels among patients with symptomatic malaria, highlighting a connection between magnesium levels and clinical status. However, given that no studies have included asymptomatic patients for comparison, the interpretation of magnesium levels across varying severities of malaria remains unclear. Based on the pooled data from two studies investigating magnesium levels in severe and nonsevere cases, the analysis revealed no statistically significant disparity in magnesium levels between the two patient groups, indicating that magnesium levels are independent of disease severity or more studies are needed to include in the meta-analysis as only two studies were available^20,39^. Furthermore, two studies^34,41^ reported increased magnesium levels in fatal cases compared with survivors. In contrast, another study^36^ found no difference. When the results from these three studies^34,36,41^ were pooled, a significant increase in magnesium levels was observed in patients who succumbed to malaria compared with survivors. This suggests that magnesium levels might have a prognostic value in gauging malaria infection severity and potential outcomes. Nevertheless, further validation is required, preferably through more extensive cohort studies.

The subgroup analysis based on the method of *Plasmodium* detection revealed a significant increase in magnesium levels in studies using microscopic detection. However, this increase was not evident in studies employing a combination of microscopy and RDT or those not specifying their detection method. While these trends suggest that the detection technique might influence the observed magnesium levels, the underlying reason for this potential effect remains uncertain. Moreover, the subgroup analysis based on the method of magnesium measurement demonstrated a significant rise in magnesium levels in studies employing atomic absorption spectrometry compared with other techniques. This suggests that the choice of measurement technique might influence the observed differences in magnesium levels between malaria patients and uninfected controls. Atomic absorption spectrometry for assessing magnesium in malaria patients could offer advantages for clinical practice over alternative methods, as it is considered a reference method for magnesium measurement because of its specificity and accuracy^48,49^. Based on the type of blood sample used for measuring magnesium, the subgroup analysis showed a significant increase in magnesium levels in studies using serum samples. In contrast, plasma studies reported no difference in magnesium levels between malaria patients and uninfected controls. While serum, plasma, or urine can typically be used for measuring magnesium in routine clinical practice^49^, the discrepancies observed might arise from other factors. As indicated by the subgroup analysis, one such factor is the technique employed for magnesium measurement. In addition, other potential contributing factors could include the stage of malaria infection, individual patient differences, nutritional status, and the presence of comorbid conditions, all of which might influence magnesium levels.

The meta-analysis provides a pivotal ground for understanding the overall trend, albeit with a high degree of heterogeneity, except for the meta-analysis of the difference in magnesium levels between fatalities and survivors. Heterogeneity flags the presence of underlying diverse factors that influence outcomes. Metaregression and subgroup analysis have indeed highlighted that the year of publication, age group, and *Plasmodium* species, among other factors, significantly influenced the pooled estimate, painting a mosaic of factors contributing to the complex relationship between malaria and magnesium levels. The methods used for malaria detection and magnesium determination significantly affected the results, calling for a standardized methodology in future studies to reduce this source of variability and garner more reliable data.

The systematic review and meta-analysis have limitations, mainly regarding the substantial heterogeneity and instability demonstrated in the sensitivity analysis. This instability requires a cautious interpretation of the results and emphasizes the necessity for larger, more robust studies to further substantiate the findings. The geographical and demographical discrepancies observed in the review underline the necessity for region-specific, demographically diverse, and larger studies investigating magnesium levels in patients with malaria. Moreover, understanding the biological underpinnings of the observed trends could be a vital area of future research, possibly guiding targeted therapeutic strategies. Moreover, given that certain groups, such as children and adults, were observed to have different outcomes in the subgroup analysis, future studies could focus on delineating the underlying mechanisms behind these differences.

## Conclusion

The systematic review and meta-analysis offered a detailed and complex landscape of the relationship between magnesium levels and malaria, influenced by various factors, including geographical regions, age groups, and clinical severity. While the meta-analysis does indicate a general trend of increased magnesium levels in patients with malaria, the substantial heterogeneity and instability of the results require more in-depth and focused research. The intricate interplay between magnesium levels and malaria beckons a multidimensional approach in future studies, encompassing a broader and more nuanced understanding, potentially paving the way to novel diagnostic and therapeutic strategies in malaria management.

### Supplementary Information

Supplementary Table S1.

Supplementary Table S2.

Supplementary Table S3.

Supplementary Table S4.

## Data Availability

All data related to the present study are available in this manuscript and supplementary files.
