# Supplementary material for: A systematic review and meta-analysis of the relationship between magnesium levels and malaria severity
Source: Sci Rep. 2024 Jan 16;14:1348. doi: 10.1038/s41598-024-51718-z (PMC10791651; doi:10.1038/s41598-024-51718-z)
Supplement: Supplementary file 1 — Supplementary Table S1. [file 41598_2024_51718_MOESM1_ESM.docx]

**A systematic review and meta-analysis of the relationship between magnesium levels and malaria severity**

Kwuntida Uthaisar Kotepui^1^, Aongart Mahittikorn^2^, Polrat Wilairatana^3*^, Frederick Ramirez Masangkay^4^, Manas Kotepui^1^*

^1^Medical Technology, School of Allied Health Sciences, Walailak University, Thasala, Nakhon Si Thammarat 80160, Thailand

^2^Department of Protozoology, Faculty of Tropical Medicine, Mahidol University, Bangkok 10400, Thailand

^3^Department of Clinical Tropical Medicine, Faculty of Tropical Medicine, Mahidol University, Bangkok 10400, Thailand

^4^Department of Medical Technology, Faculty of Pharmacy, University of Santo Tomas, Manila 1000, Philippines

*Corresponding author

Kwuntida Uthaisar Kotepui: [kwuntida.ut@wu.ac.th](mailto:kwuntida.ut@wu.ac.th)

Aongart Mahittikorn: aongart.mah@mahidol.ac.th

Frederick Ramirez Masangkay: frederick_masangkay2002@yahoo.com

Polrat Wilairatana: [polrat.wil@mahidol.ac.th](mailto:polrat.wil@mahidol.ac.th)

Manas Kotepui [manas.ko@wu.ac.th](mailto:manas.ko@wu.ac.th), Tel.: +66954392469

**Table S1. Search terms**

**General keywords**

magnesium AND (malaria OR plasmodium OR “Plasmodium Infection“ OR “Remittent Fever“ OR “Marsh Fever“ OR Paludism)

PubMed 18 September 2023

| No. | Key concept | Search terms | Results |
| --- | --- | --- | --- |
| 1. | Magnesium | magnesium[Text Word] OR magnesium[MeSH Terms] | 119,695 |
| 2. | Malaria | malaria[Text Word] OR malaria[MeSH Terms] OR plasmodium[Text Word] OR “Plasmodium Infection“[Text Word] OR “Remittent Fever“[Text Word] OR “Marsh Fever“[Text Word] OR Paludism[Text Word] | 119,462 |
| 3. | 1 AND 2 | (magnesium[Text Word] OR magnesium[MeSH Terms]) AND (malaria[Text Word] OR malaria[MeSH Terms] OR plasmodium[Text Word] OR “Plasmodium Infection“[Text Word] OR “Remittent Fever“[Text Word] OR “Marsh Fever“[Text Word] OR Paludism[Text Word]) | 148 |

Embase 18 September 2023

| No. | Key concept | Search terms | Results |
| --- | --- | --- | --- |
| 1. | Magnesium | magnesium:ti,ab,kw,de OR magnesium/exp | 201,063 |
| 2. | Malaria | malaria:ti,ab,kw,de OR plasmodium:ti,ab,kw,de OR ‘Remittent Fever’:ti,ab,kw,de OR ‘Marsh Fever’:ti,ab,kw,de OR Paludism:ti,ab,kw,de OR malaria/exp | 157,796 |
| 3. | 1 AND 2 | (magnesium:ti,ab,kw,de OR magnesium/exp) AND (malaria:ti,ab,kw,de OR plasmodium:ti,ab,kw,de OR ‘Remittent Fever’:ti,ab,kw,de OR ‘Marsh Fever’:ti,ab,kw,de OR Paludism:ti,ab,kw,de OR malaria/exp) | 366 |

Scopus 18 September 2023

| No. | Key concept | Search terms | Results |
| --- | --- | --- | --- |
| 1. | Magnesium | TITLE-ABS-KEY (magnesium) | 417,351 |
| 2. | Malaria | TITLE-ABS-KEY ( malaria OR plasmodium OR "plasmodium infection" OR "remittent fever" OR "marsh fever" OR paludism ) | 158,692 |
| 3. | 1 AND 2 | ( TITLE-ABS-KEY (magnesium) ) AND ( TITLE-ABS-KEY ( malaria OR plasmodium OR "plasmodium infection" OR "remittent fever" OR "marsh fever" OR paludism ) ) | 386 |

MEDLINE 18 September 2023

| No. | Key concept | Search terms | Results |
| --- | --- | --- | --- |
| 1. | Magnesium AND Malaria | magnesium AND (malaria OR plasmodium OR “Plasmodium Infection“ OR “Remittent Fever“ OR “Marsh Fever“ OR Paludism) | 152 |

Ovid 18 September 2023

| No. | Key concept | Search terms | Results |
| --- | --- | --- | --- |
| 1. | Magnesium AND Malaria | magnesium AND (malaria OR plasmodium OR “Plasmodium Infection“ OR “Remittent Fever“ OR “Marsh Fever“ OR Paludism) {Including Limited Related Terms}  Filter: limit to (ovid full text available and articles with abstracts and original articles) | 226 |

Nursing & Allied Health Premium 18 September 2023

| No. | Key concept | Search terms | Results |
| --- | --- | --- | --- |
| 1. | Magnesium AND Malaria | magnesium AND (malaria OR plasmodium OR “Plasmodium Infection“ OR “Remittent Fever“ OR “Marsh Fever“ OR Paludism) | 1255 |

**Abbreviations:** MeSH, Medical Subject Headings
