# Supplementary material for: A systematic review and meta-analysis of the relationship between magnesium levels and malaria severity
Source: Sci Rep. 2024 Jan 16;14:1348. doi: 10.1038/s41598-024-51718-z (PMC10791651; doi:10.1038/s41598-024-51718-z)
Supplement: Supplementary file 4 — Supplementary Table S4. [file 41598_2024_51718_MOESM4_ESM.docx]

**A systematic review and meta-analysis of the relationship between magnesium levels and malaria severity**

Kwuntida Uthaisar Kotepui^1^, Aongart Mahittikorn^2^, Polrat Wilairatana^3*^, Frederick Ramirez Masangkay^4^, Manas Kotepui^1^*

^1^Medical Technology, School of Allied Health Sciences, Walailak University, Thasala, Nakhon Si Thammarat 80160, Thailand

^2^Department of Protozoology, Faculty of Tropical Medicine, Mahidol University, Bangkok 10400, Thailand

^3^Department of Clinical Tropical Medicine, Faculty of Tropical Medicine, Mahidol University, Bangkok 10400, Thailand

^4^Department of Medical Technology, Faculty of Pharmacy, University of Santo Tomas, Manila 1000, Philippines

*Corresponding author

Kwuntida Uthaisar Kotepui: [kwuntida.ut@wu.ac.th](mailto:kwuntida.ut@wu.ac.th)

Aongart Mahittikorn: aongart.mah@mahidol.ac.th

Frederick Ramirez Masangkay: frederick_masangkay2002@yahoo.com

Polrat Wilairatana: [polrat.wil@mahidol.ac.th](mailto:polrat.wil@mahidol.ac.th)

Manas Kotepui [manas.ko@wu.ac.th](mailto:manas.ko@wu.ac.th), Tel.: +66954392469

**Table S4. Meta-regression analysis of covariates on the difference in magnesium levels between patients with malaria and uninfected controls**

| **Covariates** | ***P* value** | **tau2** | ***I^2^* (%)** | **R-squared (%)** | **Number of studies** |
| --- | --- | --- | --- | --- | --- |
| Publication years | 0.0008 | 3.326 | 98.75 | 0.00 | 9 |
| Study design | 0.9736 | 2.912 | 98.58 | 0.00 | 9 |
| Continent | 0.5068 | 3.14 | 98.43 | 0.00 | 9 |
| Age group | 0.0002 | 3.31 | 98.49 | 0.00 | 9 |
| *Plasmodium* species | 0.0133 | 2.859 | 98.56 | 0.00 | 9 |
| Clinical status | N/A | N/A | N/A | N/A | N/A |
| Diagnostic method for malaria | 0.0099 | 1.995 | 98.32 | 5.12 | 9 |
| Method for magnesium measurement | 0.0017 | 2.908 | 98.58 | 0.00 | 9 |
| Types of blood samples | 0.1030 | 3.006 | 98.53 | 0.00 | 9 |

N/A: omitted because of collinearity.
